# An Account of the Good Effects of a Mercurial Snuff in a Case of Gutta Serena

**Published:** 1793

**Authors:** R. B. Blagden

**Affiliations:** Surgeon at Petworth, in Sussex.


X. *An Account of the good Effects of a Mercurial Snuff in a Case of Gutta Serena. By Mr.* R. B. Blagden, *Surgeon at Petworth, in Sussex.*

MR. ———, aged thirty-one years, of a spare habit, and subject to scrophulous affections of the sub-maxillary glands, between four and five years ago, on a sudden, and without the smallest injury or previous indisposition, became sensible of such a defect in the sight of his right eye, that he was unable to take his favourite diversion of shooting, in the usual way: however, as the sight of the left eye enabled him to read, and to use a left-handed gun pretty successfully, he was contented; and probably would have remained so, had not that likewise began to fail:—a circumstance of which he first took notice about six weeks before he applied to me.

On the 7th of October, when I first saw him, the pupils of both eyes were contracted to as great a degree as the pupil of a sound eye is by a sudden and strong light.

The

The pupil of the left eye, on the approach of a very vivid light, ſhewed ſo ſmall an alteration as to be ſcarcely perceivable; and that of the right none at all. With the left the patient could barely ſee the capital letters which the printers call the Four Lines Pica; with the right he could only diſtinguiſh light from darkneſs.

The caſe ſeemed to me a fair one for a trial of the mercurial ſnuff recommended, and ſo ſucceſsfully uſed, by Mr. Ware, in the third volume of the Memoirs of the London Medical Society; and I, accordingly, directed the patient to take a pinch of it (prepared by mixing five grains of the hydrargyrus vitriolatus with thirty-five of the pulvis aſari compoſitus) every night. As he ſmiled at the idea of being cured by a pinch of ſnuff, I gave him two tea-ſpoonfuls of a mixture, compoſed of equal parts of tincture of valerian and compound tincture of lavender, twice a day in a cup of roſemary tea: the doſe was, afterwards, increaſed to three tea-ſpoonfuls.

On the 21ſt of October the patient could ſee the capital letters with the right eye, and could read the Four Lines Pica print with the left.— The pupils were, in their general appearance, leſs lefs contracted; and they were affected more fenfibly by the impreffion of light. The firft five or fix times of ufing the fnuff it made his nofe bleed freely, and fo long as it produced this effect, he thought he perceived the advances more ftrikingly; an additional two grains and an half of the mercurial were therefore put to the next quantity of the pulv. afari c. and the hæmorrhage from the nofe was reproduced as often as it was made ufe of.

On the 28th of October, the appearance and contraction of the pupils were natural;—the patient could read a newfpaper, and was able to fhoot correctly with his right-handed gun.

On the 18th of November, the fight of both eyes was in every refpect perfect.

*Petworth.*

December 5, 1792.

